# Thermal Behaviors, Interfacial Microstructure and Molecular Orientation of Shape Memory Polyurethane/SiO_2_ Based Sealant for Concrete Pavement

**DOI:** 10.3390/polym14163336

**Published:** 2022-08-16

**Authors:** Shuang Shi, Tao Ma, Linhao Gu, Yanning Zhang

**Affiliations:** School of Transportation, Department of Roadway Engineering, Southeast University, Southeast University Road #2, Nanjing 211189, China

**Keywords:** sealant, shape memory polyurethane, silicon dioxide, molecular orientation, thermal behaviors

## Abstract

Expansion joint failure is one of the main causes that lead to the damages of concrete pavement. The silicon dioxide/shape memory polyurethane (SiO_2_/SMPU) is a new kind of sealant which can use its shape memory performance to adapt to the width of the expansion joint with the change of pavement temperature, and it can effectively prolong the service life of the pavement and reduce maintenance costs. In this study, the effects of programming and the addition of SiO_2_ particles to the thermodynamic properties of the specimens were detected using differential scanning calorimetry (DSC), the optimal shape memory programming temperature of which is 72.9 °C. Combined with scanning electron microscopy (SEM) and shape memory effect test, the particles are evenly distributed between the two phases, and the shape fixation rate (R*_f_*) of 98.15% and the shape recovery rate (R*_r_*) of 97.31% show that the composite has a good shape memory effect. Fourier transform infrared spectroscopy (FTIR) and dynamic infrared dichroism illustrate the change of the hydrogen bond of soft and hard segments with the SiO_2_ particles in the shape memory cycle, revealing the optimal shape memory programming process. This study provides an insight into the reinforcement mechanism of SiO_2_ nanoparticles in SMPU matrix and verify whether it can meet the engineering requirements of expansion joints when used as a sealant of concrete pavement.

## 1. Introduction

Cement concrete pavement has become one of the main paving types of high-grade pavement due to its high strength, good stability and long service [1]. Most of the cement concrete pavement are jointed cement roads. The joints are some of the weakest parts of cement pavement, which is the main factor leading to cement pavement damage. The sealing effect and durability of sealant directly affect the waterproofness, tightness and smoothness of cement pavement joints, and further affects the life span of the pavement, the comfort level of driving, and maintenance and repair costs [2]. However, the cement pavement sealant at present is difficult to adapt to the environmental conditions of the joint and the cyclic expansion and contraction of the joint width. Further, there is no self-healing function, which cannot meet the requirements for the use and development of the cement pavement. Liu et al. [3] prepared asphalt-based sealant with five different additives and found that the mixture performance of asphaltic plug joints can be influenced by proper number of additives. Li et al. [4] studied a novel adhesive strength method to define the silicone sealants’ adhesive strength as self-leveling or not.

Nowadays, many kinds of sealants have been used to seal expansion joints on concrete pavement, such as hot-poured, cold-poured or preformed. Due to the damages of the existing sealants (cohesive and adhesive failure), more and more new materials are being researched [5]. Among them, Shape memory polyurethane (SMPU), as a kind of important sealant materials, can maintain a temporary shape at a specific temperature and recover to the original shape after stimulated by light, humidity, radiation, temperature or magnetic field, etc. [6]. SMPUs show such advantages as large deformation, convenient programming, easy adjustment of shape responsive temperature, light weight, corrosion resistance, etc. [7].

However, there is a thermodynamic incompatibility between soft and hard segments in SMPU due to their different chemical constituents. This incompatibility causes the segregation between the soft and hard segments in SMPU, and hard segments are mutually aggregated. As a result, two phase structure is formed in SMPU, including a soft segment region (soft segment phase) and a hard segment region (hard segment phase), which is the microphase separation in SMPU [8].

At the same time, the chemical connection between soft and hard segments reduces macro phase separation in SMPU. The sizes of hard segments in SMPU are usually from 5 nm to 100 nm with fibrous, spherical, cylindrical and plate shapes. In addition to covalent interactions, there are non-covalent interactions between the hard segment chain and/or the hard segment region in SMPU, such as hydrogen bonding, dipole interaction, etc. [9]. Under the action of non-covalent bonds, the independent hard segment regions are further aggregated and assembled, resulting in an increase in the size of hard segment region even to the micron level. Since there are different structures and performances between soft and hard segments, the two-phase structure due to phase separation brings local micro-nano structures with different properties in SMPU [10].

It was known from the shape memory mechanism of SMPU that the phase separation microstructure is constantly changed during the shape memory effect cycle [6]. Zhang et al. [11] found that the SMP could quickly return to its permanent shape when the temperature exceeded the transition temperature by pulling the hard segments by soft segments in a phase separation structure during the shape memory cycle. Wongsamut et al. [12] investigated the effect of hard-soft segment ration on adhesion properties, indicating that distinct phase structure partitions in an unambiguous phase-separated structure have an impact on the performance of the SMPU.

Additionally, Priyanka et al. [13] investigated the shape memory properties of SMPU based composites with its detailed chemical and thermo-mechanical characterization, indicating that effects of the original shape of SMPU on the degree of phase separation were related to the distance between hard segment microdomains and the space required for hard segment crystallization. In addition, the programming, fixing and recovery are three necessary steps to characterize shape memory performance of SMPU [14]. Morozov et al. [15] point out that the surface free energy and the length of the soft and hard segments change with the stretch direction of the temporary pattern during the shape memory cycle. Gonzalez et al. [16] observed similar changes in morphology and orientation of hard segment domains when SMPU was stretched.

In our previous study [17], we found that soft segment molecular chains in SMPU was oriented after the uniaxial stretching, and the phase structure and microscopic morphology of hard and soft segment microdomains were changed to affect mechanical properties of SMPU. However, the filling effects of SiO_2_ nanoparticles on thermal behaviors, shape memory effect, interaction among microphase structure and molecular orientation of porous SMPU matrix were not discussed.

It was found that the addition of particle reinforcement could enhance mechanical performance and shape recovery force of SMPU [18]. Among those reinforcement particles of SMPU, the molecular state of SiO_2_ nanoparticle is a three-dimensional chain structure, which interacts with the electron cloud of SMPU matrix. A network structure is formed in SMPU by the effective entanglement based on physical reaction, which is conductive to improve the mechanical properties, aging resistance and thermal stability of SMPU. Therefore, properties of SiO_2_/SMPU nanocomposite were paid more and more attention [18].

Shi et al. [19] prepared SiO_2_/SMPU composite and found that it had great shape memory effect and mechanical properties when SiO_2_ particle content was 15%. Zhang et al. [20] pointed out that nano SiO_2_ particles on the surface could effectively enhance UV resistance, thermal stability and interfacial shear strength. Yousefi et al. [21] optimized the effective parameters of surface tension and durability of synthesized coatings and provided a simple preparation method of SiO_2_/SMPU nanocomposite. Huang et al. [22] used the IR to study the hard and soft segments in a shape memory cycle and reported that the mechanism of change in soft and hard segments motion.

Currently, it is noted that influences of SiO_2_ nanoparticle on thermal stability, shape memory performance, interfacial microphase structure and molecular orientation of SMPU were rarely investigated. Further, the filling effects of SiO_2_ nanoparticles on molecular orientation of porous SMPU matrix as well as the interaction between SiO_2_ nanoparticles and SMPU were seldom reported. As a result, it is difficult to reveal the reinforcement mechanism of SiO_2_ nanoparticles and shape memory mechanism of SiO_2_/SMPU nanocomposite. The objective of this study is to better understand thermal behavior and shape memory effect of SiO_2_/SMPU nanocomposite used as a sealant for expansion joint on concrete pavement, as well as characterize changes in interfacial microstructure and molecular orientation of SiO_2_/SMPU during a shape memory cycle. This provides an insight into the reinforcement mechanism of SiO_2_ nanoparticles on SMPU matrix and the shape memory mechanism of SiO_2_/SMPU nanocomposite, which further explain that programmed sealant can better adapt to the engineering application requirements of expansion joints in a working environment that meets the road temperature.

In this study, SiO_2_/SMPU nanocomposite was synthesized by the in-situ polymerization method. The glass transition temperature (T_g_) and the phase transition temperature (T_trans_) of SiO_2_/SMPU nanocomposite was determined by DSC tests, further confirming whether the T_trans_ meets the working requirements. R*_f_* and R*_r_* were calculated to evaluate shape memory effect of SiO_2_/SMPU nanocomposite. Then, FESEM was used to observe the microscopic morphology changes of original, programmed and recovered SiO_2_/SMPU nanocomposites, respectively. After that, FTIR and dynamic infrared dichroism tests were conducted to discuss microstructure changes at the interface between SiO_2_ nanoparticles and SMPU matrix, as well as molecular orientation changes of original, programmed and recovered SiO_2_/SMPU nanocomposite, revealing the reinforcement mechanism of SiO_2_ nanoparticles on SMPU matrix and the shape memory mechanism of SiO_2_/SMPU nanocomposite. Generally, the highest temperature of pavement in summer as the response temperature of SiO_2_/SMPU nanocomposite sealant, it realizes the self-healing of the sealant by recovering to the original shape in presence of an external stimuli, which greatly prolongs the service life and reduces the maintenance cost.

## 2. Experimental

### 2.1. Synthesis of SiO_2_/SMPU Nanocomposite

#### 2.1.1. Raw Materials

Main materials used in this study include polyadipate-1,4-butanediol diol (PBAG, Mn = 2000, industrial grade, Asahikawa Chemical Co., Ltd., Suzhou, China), toluene diisocyanate (TDI, chemically pure, TCI (Chemical Industry Development Co., Ltd., Shanghai, China)), 1,4-butanediol (BDO, analytical grade, Sinopharm Chemical Reagent Co., Ltd., Shanghai, China), and SiO_2_ powder with the particle size of 16 nm (Changtai Chemical Plant, Shandong, China).

#### 2.1.2. Synthesis of Samples

SiO_2_/SMPU nanocomposite was prepared through uniformly dispersing 15% by volume of SiO_2_ nanoparticles in SMPU matrix. SiO_2_/SMPU nanocomposite was synthesized by in-situ polymerization. The calculated amount of PBAG was placed in a 250 mL 4-neck round-bottom flask, which was equipped with a thermometer, a mechanical stirrer, and nitrogen inlet and outlet tubes. PBAG was dehydrated at 120 °C for 1.5 h under vacuum environments (vacuum degree > 0.095 MPa). When the temperature was lowered to 80 °C, a calculated amount of TDI was added. Under the protection of nitrogen, the temperature was maintained at 80 °C for 2 h to obtain SMPU prepolymer.

**Figure 1:**
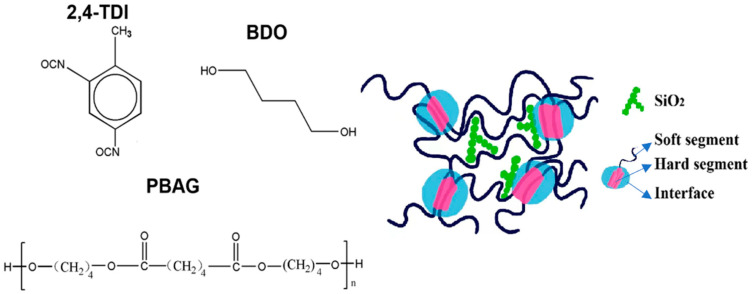
Schematic diagram of chemical structures of molecular segments in SiO_2_/SMPU nanocomposite.

Then SiO_2_ nanoparticles were added into the prepolymer. The mixture was stirred using a mechanical stirrer for about 10 min for uniform dispersion of SiO_2_ particles. After that, when the temperature was lowered to 70 °C, the required amount of chain extender of BDO was added dropwise into the mixture which was rapidly stirred for 30 min. The synthesized SiO_2_/SMPU nanocomposite was immediately injected into polytetrafluoroethylene molds where the homogeneous mixture was cooled to room temperature and cured. Thus, a SiO_2_/SMPU nanocomposite sample was obtained after demolding. Finally, this sample was subjected to various property characterizations. The schematic diagram of chemical structures of molecular segments in SiO_2_/SMPU nanocomposite was shown in Figure 1.

### 2.2. Characterization Method

#### 2.2.1. DSC Test

DSC (204F1 type, Netzsch, Germany) was utilized to discuss the thermal behaviors of soft and hard segments and determined the transition temperature (Ttrans) of SiO_2_/SMPU composite. About 15 mg SiO_2_/SMPU sample was heated from −40 °C to 200 °C at a heating rate of 10 °C/min in the nitrogen environments. After that, SiO2/SMPU sample was cooled from 200 °C to −40 °C at a cooling rate of 20 °C/min, and then was heated to 200 °C at the heating rate of 20 °C/min. DSC test results in the second heating process were obtained to analyze thermal properties of SiO_2_/SMPU composite through eliminating the thermal history. This experiment was repeated three times.

#### 2.2.2. Shape Memory Test

**Figure 2:**
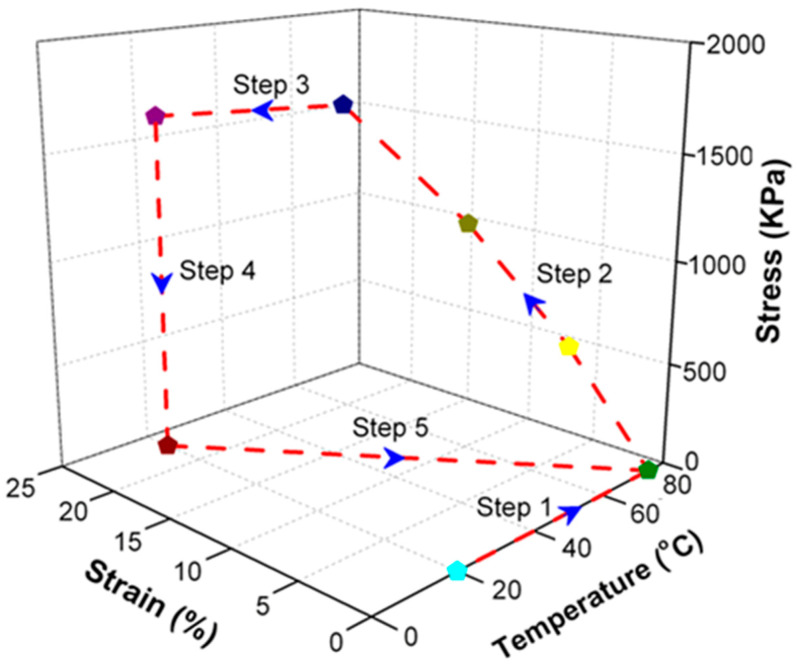
Schematic diagram of a five-step thermodynamic cycle for programming and recovery of SiO_2_/SMPU nanocomposite. (The red dotted line completes the shape memory cycle along the blue arrow, the starting point (light blue symbol) is heated to 80 (green symbol), loaded to 25% respectively (yellow, grass green and dark blue indicate different loading stresses), purple symbol means cooling to room temperature, and dark red means room temperature unloading load).

To endow SiO_2_/SMPU nanocomposite with shape memory effect, it was usually subjected to a typical five-step thermodynamic cycle for programming and free recovery as shown in Figure 2. The uniaxial tensile programming using a truss fixture and shape recovery were described in our previous study [17].

To endow SMPU and SiO_2_/SMPU composite with shape memory effects, it was usually subjected to a typical shape memory cycle called programming and recovery.

To evaluate influences of SiO_2_ contents on shape memory effects of SMPU, the prepared pure SMPU and SiO_2_/SMPU specimens which were first machined into dog bone fixed on the fixture separately, and then placed in a heating chamber without any loading. Meanwhile, the chamber was heated to T_trans_ and held for 20 min to make the temperature distribute uniformly in the sample.

On this basis, the sample was taken out after programming for 2 h, and cooled to room temperature. A continuous load is required during the entire process of programming and cooling, then the load was removed and put the sample at room temperature for 24 h.

Finally, the sample stood for 24 h was put back without any loading in the chamber from the room temperature to T_trans_ to recovery for two hours. The pre-strain of programmed specimen reached 25%, and the shape fixity ratio (R*_f_*) and shape recovery ratio (R*_r_*) were calculated to evaluate shape memory effects, which were expressed as follows.

R*_f_* = (*l*_2_ − *l*_0_)/(*l*_1_ − *l*_0_) × 100% (1)

R*_r_* = (*l*_2_ − *l*_3_)/(*l*_2_ − *l*_0_) × 100% (2)

where, *l*_0_, *l*_1_, *l*_2_ and *l*_3_ are the tagged middle lengths of original, programmed with loading at room temperature, programmed without loading at room temperature and recovered specimens, respectively.

#### 2.2.3. SEM Observation

Morphology changes of original, programmed and recovered SiO_2_/SMPU samples were characterized using FESEM (JSM-7600F, JEOL, Tokyo, Japan), respectively. Samples were first fixed on an aluminum stub and sputtered with gold under vacuum conditions. The 10 mm × 10 mm × 10 mm cross-sections samples cut from the pure SMPU and SiO_2_/SMPU specimens in different states were first fixed on an aluminum stub and further sputtered with gold under vacuum conditions. Then, the sample chamber was opened to place samples. Finally, the morphologies of the samples were observed using FESEM.

#### 2.2.4. FTIR Test

To study the changes of molecular segments during a five-step thermodynamic cycle, FTIR tests were conducted on the original, programmed and recovered SiO_2_/SMPU samples, respectively. The sample was prepared by KBr tableting method, and a small amount of KBr crystal was thoroughly ground using an agate mortar. Then, the tested solid sample was added by 5%, and mixed until the mixture was uniform. Finally, the powder was transferred to a metal mold, and placed in a hydraulic press for tableting, and then FTIR tests were performed.

#### 2.2.5. Dynamic Infrared Dichroism Test

To further verify the changes in molecular orientation degree of prepared SiO_2_/SMPU nanocomposite in the original, programmed and recovered states, respectively, Fourier transform infrared spectrometer (Nicolet 6700, Thermo Fisher Scientific, Waltham, MA, USA,) was used to perform dynamic infrared dichroism tests. The spectral resolution was 4 cm^–1^, and each spectral line was the average of 32 scanning values. SiO_2_/SMPU samples were ground to light permeation, respectively. The orientation function value (*f*) of the particular represented bond was calculated according to the following Equation (3) to further quantitatively analyze shape memory property of SiO_2_/SMPU nanocomposite [23].

*f* = (R − 1)/(R + 2) × (2/(3cos2*α* − 1) (3)

where, *f* was orientation function, *α* was the angle between the direction in oscillating transition moment and molecular chain, and R was the dichroic ratio of measured particular absorption band, and its value was calculated based on Equation (4).

R = (A**_∥_**)/(A**_⊥_**) (4)

where, A**_∥_** and A**_⊥_** represented the infrared spectrum absorption intensities which were parallel and perpendicular to the stretching direction of SiO_2_/SMPU specimen during the programming, respectively.

## 3. Results and Discussion

### 3.1. Thermal Behaviors

**Figure 3:**
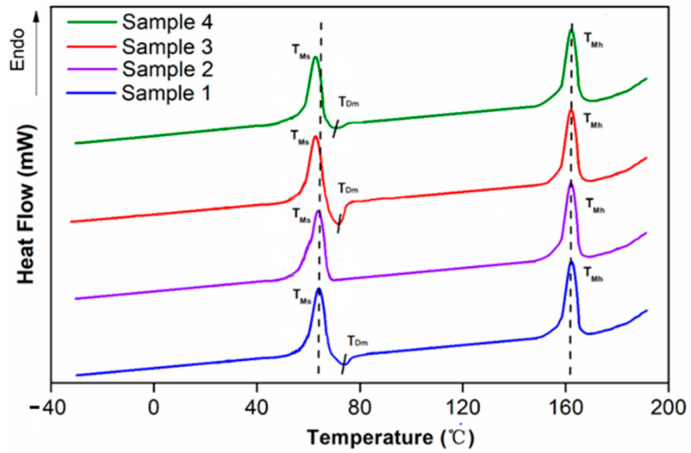
DSC thermograms of SMPU (sample 1), programmed SMPU (sample 2), SiO_2_/SMPU (sample 3) and programmed SiO_2_/SMPU (sample 4).

In order to be able to study the effects of programming and the addition of the SiO_2_ particle to the thermodynamic properties of the specimens, and to determine the T_trans_ of the SiO_2_/SMPU composite for further discussion of its shape memory effect, the pure SMPU and SiO_2_/SMPU composite before and after programming were both tested by DSC. The results are shown in Figure 3.

It can be deduced from Figure 3 that the samples of pure SMPU and SiO_2_/SMPU composites from before and after programming tend to have no obvious step-shape curve, it is difficult to confirm the T_g_ of the specimens. However, there are obvious endothermic peaks near 60 °C, which indicate the melting temperature of the soft segments (T_Ms_). Because the SMPU is a kind of typical microphase separation structure polymer [18], when the temperature is lower than its T_Ms_, the molecular kinergety of soft phase in SMPU is too low to overcome the rotation barriers within the main chains [24]; therefore, the motion of chain segments does not occur. In addition, with the exception of the pure SMPU sample after programming, there are different degrees of exothermic peaks at T_Dm_ (around 72.9 °C), which can be seen as a phase separation temperature which short hard segments segregate from the mixed phase and aggregate into the micro-hard phase [25]. As the temperature rises to the melting point of hard segments (T_Mh_), which shown obvious endothermic peaks near 164.5 °C, the specimens gradually fail to maintain its morphology, it is indicated that the soft segments provide elastomeric features, whereas the hard segments act as reinforcing components and provide dimensional stability [26].

Compared to the pure SMPU and SiO_2_/SMPU composites, the T_ms_ shift to the left with the SiO_2_ particles adding, this is due to the fact that the addition of SiO_2_ affects the symmetry of the soft segment molecular chain, and the hydrogen bonds forming with the soft segment increase the binding force among the soft segments [27]. As crystallinity decreases, the T_ms_ both decreases, Simultaneously, the exothermic peak area of the SiO_2_/SMPU composite is slightly larger than pure SMPU, which is due to the added SiO_2_ particles form hydrogen bonding with the soft segment, and with some hard segments, in order for some short hard segment molecular chains to be pulled by hydrogen bonds and separated from the mixed phase [28]. However, the hard segment acts as a stationary phase—some ordered long molecular segments are not susceptible to SiO_2_, so that the peak end of the melting endotherm of the hard segment does not move.

When the programming is performed at a temperature higher than that which corresponds with the exothermic peak of 72.9 °C, the hydrogen bonds between the soft and hard segments are reduced during the heating process as a result. Without the strong restriction of the hard segments, the soft segments can possess greater agility and lower stiffness [28], and that leads to programming at around 72.9 °C being better to extend the soft segment along the stretching direction. This can be seen from the comparison between the pure SMPU and the composite material in Figure 3, both before and after programming. As a result, 72.9 °C can be seen as the T_trans_.

Based on the premise that the T_Mh_ are almost constant, the T_Ms_ shifts slightly to the left, and at the same time, the exothermic peak which represents the phase separation movement gradually disappears. Compared with the pure SMPU and the composite material after programming, it can be observed that due to the addition of SiO_2_ particles, the microphase separation of the material is hindered, which means that the soft segment cannot be completely distorted and requires further verification by the shape memory effect.

### 3.2. Shape Memory Effect

**Figure 4:**
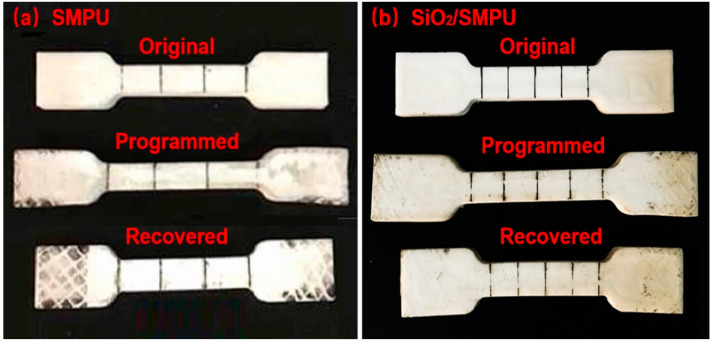
Length comparison among original, programmed and recovered specimens of (**a**) pure SMPU and (**b**) SiO_2_/SMPU nanocomposite.

Figure 4 shows the comparison photographs of original, programmed and recovered pure SMPU and SiO_2_/SMPU specimens, respectively. Test results of specimen lengths, calculated R*_f_* and R*_r_* of pure SMPU and SiO_2_/SMPU nanocomposite are summarized in Table 1.

It is seen from Figure 4 and Table 1 that programmed SMPU and SiO_2_/SMPU specimens are elongated in the stretching direction. Then, when the temperature is raised to T_trans_ °C and maintained for 30 min, the two specimens are almost restored to their original lengths after the free recovery process. This is because the elastic strain energy is prestored in the programmed SMPU and SiO_2_/SMPU specimens during the programming, thus providing a driving force for the thermally induced SMPU and SiO_2_/SMPU specimens to restore to their original shapes.

As shown in Table 1, the average values of R*_f_* and R*_r_* of SMPU are 100%; however, the average values of R*_f_* and R*_r_* of SiO_2_/SMPU nanocomposite are 98.15% and 97.31%, respectively, which are slightly less than 100%. The reason for this is that the mixture and microphase separation are directly affected by the crystallization of pure SMPU matrix. However, the addition of SiO_2_ nanoparticles lowers the orderliness of microcrystals and paracrystals in SMPU. Crystal imperfections are increased to slightly lower the shape memory effect of SiO_2_/SMPU nanocomposite [29]. Another reason is that the movements of molecular segments and SiO_2_ nanoparticles cause the damages and deformation of formed stable network in SMPU during the programming and recovery. However, the prepared SiO_2_/SMPU nanocomposite still shows an excellent shape memory performance.

### 3.3. Microstructure Changes

**Figure 5:**
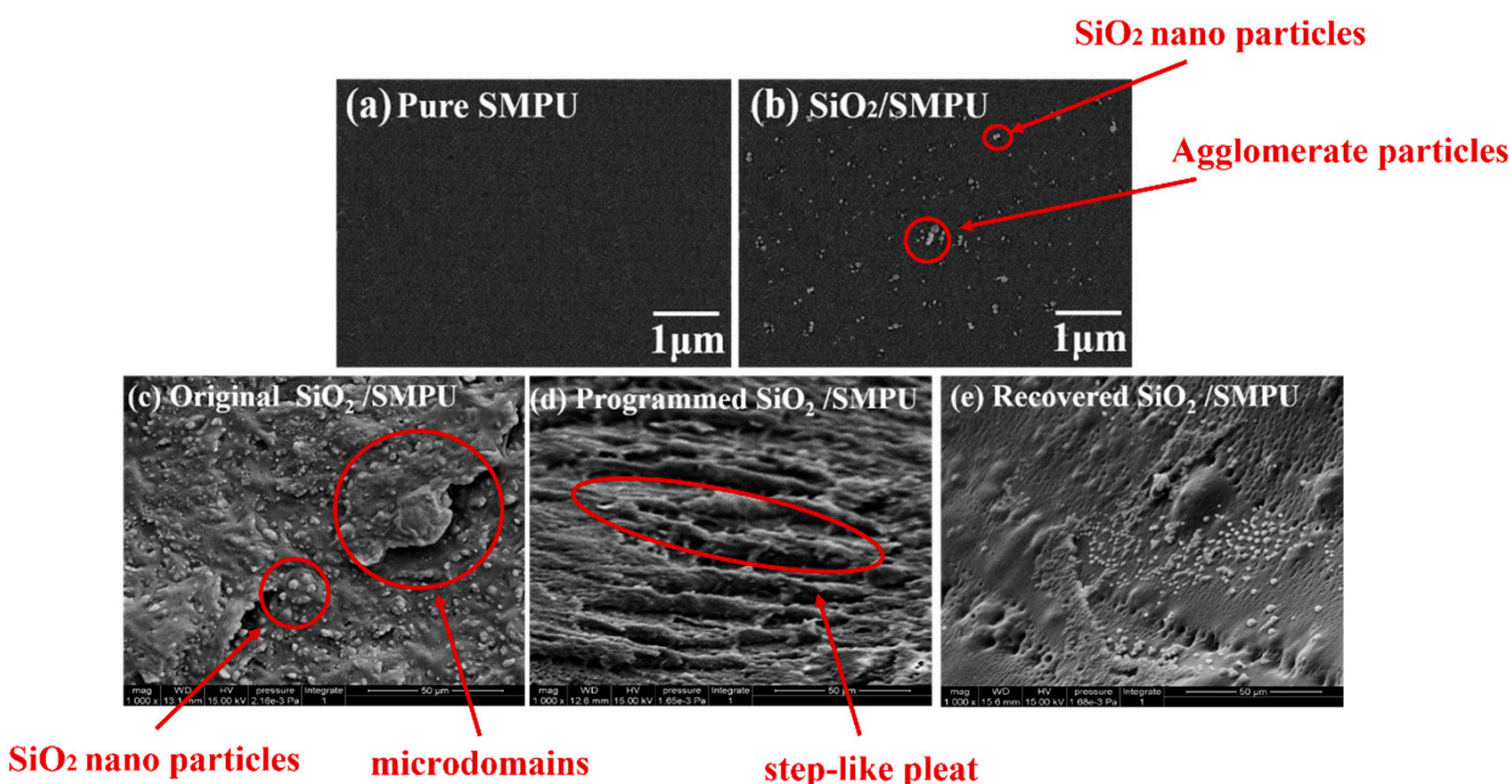
SEM images of (**a**) pure SMPU, (**b**) SiO_2_/SMPU, original (**c**), programmed (**d**) and recovered (**e**) SiO_2_/SMPU nanocomposites.

In order to discuss the distribution of SiO_2_ nanoparticles and the microstructure differences among original, programmed and recovered SiO_2_/SMPU nanocomposites, the Figure 5 shows the SEM images of original pure SMPU, original, programmed and recovered SiO_2_/SMPU nanocomposites.

The microstructure of the pure SMPU and SiO_2_/SMPU nano composite can be found in Figure 5a,b, indicating that SiO_2_ nanoparticles is more evenly distributed in the two-phase structure of SMPU. Further compared between Figure 5b,c, the different size SiO_2_ nanoparticles are distributed at the specimen surface where some of the protrusions are present. These apparent protrusions are due to the aggregation of soft segments and hard segments to form respectively their own microdomains, indicating that SiO_2_/SMPU shows crystallization [30]. The SiO_2_ nanoparticles with different sizes also indicate that the appropriate filling amount can be formed by electrostatic or van der Waals force to form a moderately sized particle size, thereby effectively filling part of the pores formed during the SMPU reaction. [31]

As shown in Figure 5d, the programmed SiO_2_/SMPU sample has been subjected to unidirectional horizontal stretching at T_trans_ °C, and molecular segments are oriented along the stretching direction. The molecular chain length is increased under the influence of micro-Brown motion. Some step-like pleats are seen on the surface of programmed SiO_2_/SMPU. This is because there are obvious differences in the modulus between soft and hard segments, and their borne loads are different. Additionally, it is worth noting that some segments are damaged, suggesting that there are microscopic losses during the five-step thermodynamic cycle.

From Figure 5e, the step-like pleats caused by the programming are almost not found on the surface of recovered SiO_2_/SMPU sample. This is because molecular chains in soft segments are restored to original curled states after experiencing a free recovery process at T_trans_ °C. As a result, the step-like pleats on SiO_2_/SMPU sample disappear.

### 3.4. Interactions among Microphase Structures

To further discuss the interaction among microphase structures of SiO_2_/SMPU nanocomposite during a five-step thermodynamic cycle (see Figure 2), FTIR tests are conducted on original, programmed and recovered SiO_2_/SMPU samples, respectively, characterizing the changes in hydrogen bond and interaction among microphase structures. Consequently, the influence of SiO_2_ nanoparticles on the shape memory performance of SMPU during a five-step thermodynamic cycle is discussed.

#### 3.4.1. Characteristic Functional Groups in Soft and Hard Segments

**Figure 6:**
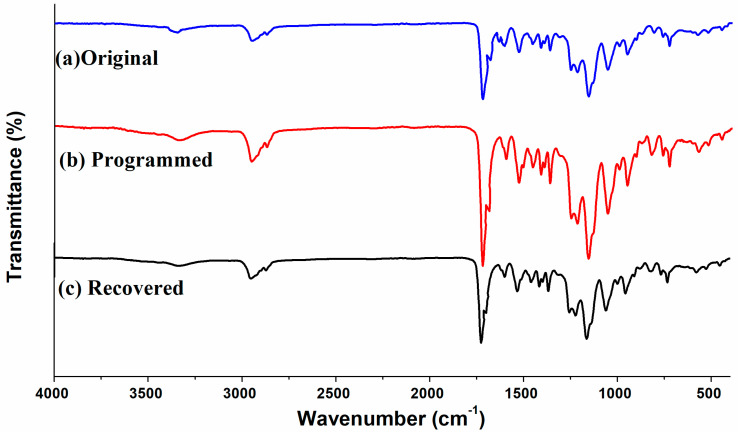
FTIR spectra of original, programmed and recovered SiO_2_/SMPU nanocomposites.

Figure 6 shows FTIR spectra of original, programmed and recovered SiO_2_/SMPU samples, respectively.

As shown in Figure 6, the characteristic absorption peaks of original, programmed and recovered SiO_2_/SMPU samples are similar, indicating no new chemical compositions are generated during a five-step thermodynamic cycle [32]. Among them, it is found from Figure 6) that the absorption peak at 1725 cm^−1^ is attributed to the stretching vibration of C=O in the freely vibrating amide. However, the relatively weak stretching vibration peaks of hydrogen-bonded C=O in the amide appear at 1709 and 1702 cm^−1^. This is because hydrogen bonds are formed owing to the conjugation of lone-pair electrons on N atoms to some C=O groups [33,34].

At the same time, the absorption peaks at 670 and 1100 cm^−1^ are due to the out-of-plane deformation vibration of –NH and the stretching vibration of C–O, respectively. A relatively strong stretching vibration peak of –NH appears at around 3338 cm^−1^. This indicates that original SiO_2_/SMPU sample includes –CO–NH and –NH–CO–O–, which are typical functional groups of SMPU.

Additionally, the characteristic absorption peaks at 2943 and 1597 cm^−1^ are ascribe to the stretching vibration of –CH_2_ and C=C on the benzene rings [35]. The –NCO group in molecular structure of TDI is not symmetrical, and the activity of 2–NCO and 4–NCO is different, and 4-NCO may be reacted to form a high molecular weight segment, covering on 2–NCO [21]. However, no stretching vibration peaks of –N=C=O groups appear at 2250–2275 cm^−1^, suggesting that SMPU is successfully synthesized and the reaction is fully completed [21]. Additionally, the absorption peaks at 799 and 1093 cm^−1^ are attributed to the symmetric stretching vibration of Si–O and the anti-symmetric stretching peak of Si–O–Si, respectively.

As shown in Figure 6, FTIR changes of original, programmed and recovered SiO_2_/SMPU samples are not obvious, in which the characteristic absorption peak of programmed sample is the weakest. The reasons for this are that prepared SMPU matrix is foamed material with a lot of pore structures. SiO_2_ nanoparticles are filled in the pores, and well wrapped by SMPU matrix so that it is difficult to detect and show obvious characteristic absorption peaks of SiO_2_ nanoparticles. In particular, molecular chains in soft segments are oriented after the programming process of SiO_2_/SMPU.

During the movement of reversible soft segments, since some SiO_2_ nanoparticles are physically entangled with soft segments, SiO_2_ nanoparticles are also moved to further fill in the pores which have not been completely filled during the preparation of SiO_2_/SMPU nanocomposite. Therefore, the above SiO_2_ nanoparticles are further wrapped by SMPU matrix. This indicates that SiO_2_ nanoparticles are more uniformly dispersed and encapsulated in the programmed SiO_2_/SMPU.

**Figure 7:**
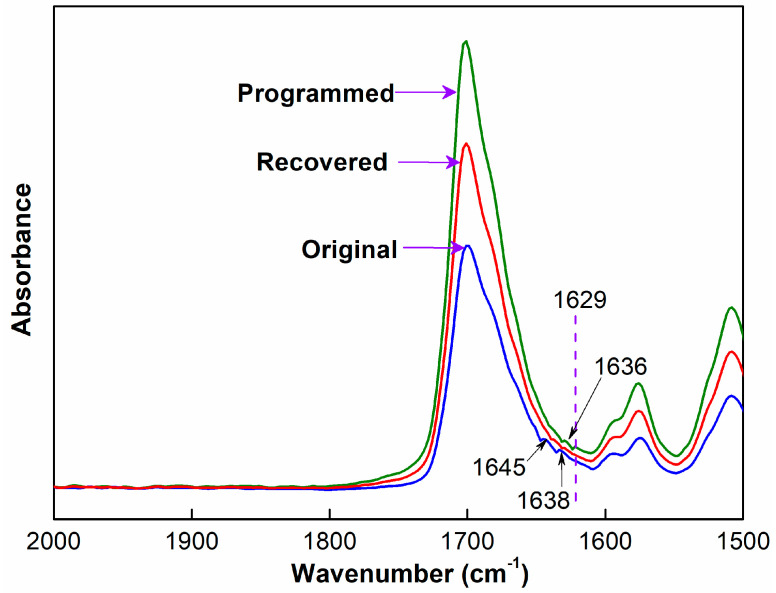
Characteristic absorption peaks of hydrogen bonds on FTIR spectra of original, programmed and recovered SiO_2_/SMPU samples.

To further characterize orientation changes of soft and hard segments in original, programmed and recovered SiO_2_/SMPU samples, respectively, characteristic absorption peak changes of hydrogen bonds of amide group (–HNCO–) on FTIR spectra are discussed as shown in Figure 7.

It is observed from Figure 7 that although FTIR spectra of original, programmed and recovered SiO_2_/SMPU samples are basically similar, the intensities of stretching vibration peaks at 1645 and 1638 cm^−1^ become weaker, and two new stretching vibration peaks at 1636 and 1629 cm^−1^ appear on FTIR spectra of programmed SiO_2_/SMPU sample.

Among them, the absorption peaks at 1645 and 1636 cm^−1^ are assigned to the short-range disordered and long-range disordered hydrogen bonds between soft and hard segments, respectively [33]. However, the absorption peaks at 1638 and 1629 cm^−1^ are attributed to the orderly hydrogen bonds in hard segments of SMPU [34]. The possible reason is the orderliness of molecular segments is improved during the uniaxial tension programming of SiO_2_/SMPU specimen, thereby increasing the number of hydrogen bonds, and changing synchronously the hydrogen bond type [35].

As shown in Figure 7, the two new stretching vibration peaks at 1636 and 1629 cm^−1^ almost disappear on FTIR spectra of recovered SiO_2_/SMPU, while the intensities of stretching vibration peaks at 1645 and 1638 cm^−1^ are higher than that of programmed SiO_2_/SMPU, but lower than that of original SiO_2_/SMPU. This is due to the fact that orientated molecular segments are restored to freely curled states during the recovery of SiO_2_/SMPU nanocomposite. Thus C=O groups are restored to free state because of some hydrogen bond cleavages, leading to the increase in stretching vibration peak intensity of C=O in the freely vibrating amide at 1680 cm^−1^ [36].

#### 3.4.2. Hydrogen Bond Changes

**Figure 8:**
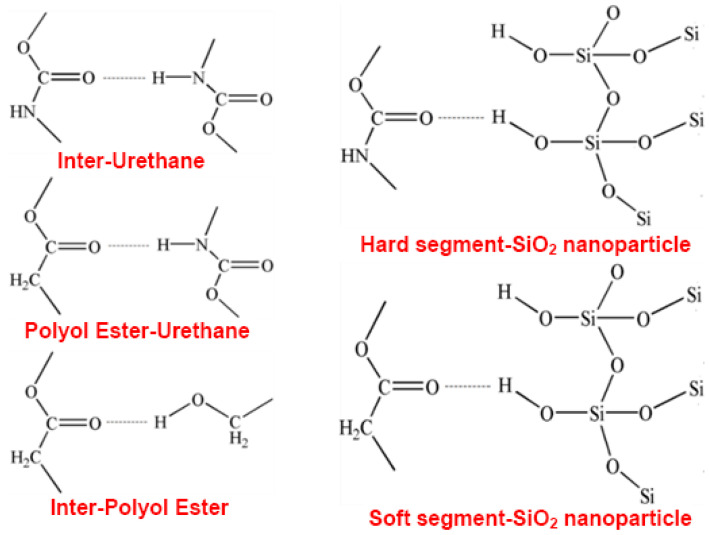
Hydrogen bond types in SiO_2_/SMPU nanocomposite.

To further study the relationship among intermolecular or intramolecular hydrogen bonds of characteristic functional groups, the hydrogen bond changes of characteristic functional groups in hard segments are discussed according to FTIR spectra of SiO_2_/SMPU samples. Figure 8 shows hydrogen bond types in SiO_2_/SMPU nanocomposite [37].

It is seen from Figure 8 that there are three proton donors in SiO_2_/SMPU nanocomposite system, including N–H in urethane, –OH in SiO_2_ and –OH in PBAG, respectively, as well as two proton acceptors, namely C=O in urethane and C=O in 1,4-butanediol ester [38]. The urethano exists mainly at the phase interface between soft and hard segments, and the hydrogen bond among urethane groups provide the morphological change of phase interface region [39].

The stretching vibration regions of urethane N–H and C=O groups are commonly used to analyze the nature of the formed hydrogen bonds [40]. The hydrogen bond indices of the N–H (R_N–H_) and C=O (R_C =O_) groups in the SMPU are calculated using Equations (5) and (6). Table 2 shows the results of hydrogen-bonding indexes of N–H and C=O functional groups in SMPU at different tensile strain levels during the programming of SiO_2_/SMPU [41].

R_N–H_ = A_3338_/A_2943_ (5)

R_C=O_ = A_1709_/A_1725_ (6)

where, A is the absorbance of characteristic band.

As shown in Table 2, as the stretching strain is increased from 0% to 25%, R_N-H_ of programmed SiO_2_/SMPU is decreased, while R_C=O_ is increased when compared with that of original SiO_2_/SMPU. The reason for this is, on the one hand, some new hydrogen bonds are formed between SiO_2_ nanoparticle and molecular chain segments as soft segments are stretched during the programming of SiO_2_/SMPU.

On the other hand, some urethane segments, which are dispersed in the curled soft segments of original SiO_2_/SMPU and don’t participate in the formation of hydrogen bonds, are orderly rearranged as the stretching strain is increased during the programming of SiO_2_/SMPU. Therefore, these urethane segments are combined with hard segments to form hydrogen bonds, entering into hard segments and bringing ordered structures in hard segments [42].

At the same time, some hydrogen bonds formed by –NH groups in urea and C=O groups in PBAG are reduced. This is because some original hydrogen bonds are broken as molecular chains move during the orderly rearrangement of soft segments from naturally curled state to ordered structure along the stretching direction. However, the hydrogen bonds between –NH and C=O groups of amides in hard segments are increased. This is due to the fact that –NH groups move toward hard segments and combine with free-vibrating C=O groups of amides in hard segments to form hydrogen bonds as the stretching strain is increased [43].

Thus, the total number change of hydrogen bond in –NH group is not obvious, but the total number of hydrogen bonds of C=O groups in hard segments is increased. As the hydrogen bond interaction among hard segments is enhanced, it is more conducive to the separation of microphase structure, thus effectively optimizing its mechanical properties [44]. The characteristic absorption peak of –NH group is not changed obviously in the three states of SiO_2_/SMPU nanocomposite, while the stretching vibration peaks of hydrogen-bonded C=O groups in the amide move towards to lower wavenumber at 1645 and 1638 cm^−1^ as the hydrogen bond number is changed. These are consistent with the test results of FTIR.

### 3.5. Molecular Orientation Changes during a Shape Memory Cycle

**Figure 9:**
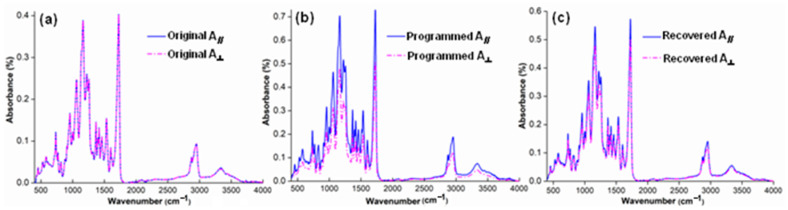
Fourier infrared polarized spectra of (**a**) original, (**b**) programmed and (**c**) recovered SiO_2_/SMPU samples.

To further discuss the shape memory mechanism of SiO_2_/SMPU nanocomposite during a five-step thermodynamic cycle (see Figure 2), Fourier infrared dichroism tests are conducted to better understand the molecular orientation changes of original, programmed and recovered SiO_2_/SMPU samples, respectively. Figure 9 presents Fourier infrared polarized spectra of original, programmed and recovered SiO_2_/SMPU samples.

It is known from Figure 6 that the absorption peak at 2943 cm^−1^ is due to the presence of -CH_2_ group in soft segments, thus which is selected to characterize the molecular orientation changes of soft segments in SiO_2_/SMPU sample [45]. The absorption peaks at 1645 and 1680 cm^−1^ are attributed to the existence of hydrogen-bonding -CONH- group at the amide interface and -CONH- group in hard segments, respectively, which also selected to characterize the molecular orientation changes on the interface between soft and hard segments [46]. Finally, the absorption peak at 3338 cm^−1^ is owing to the appearance of –NH group in hard segments, thus which is selected to show the molecular orientation changes of hard segments in SiO_2_/SMPU sample.

It is observed from Figure 9 that the characteristic absorption peak intensity of programmed SiO_2_/SMPU sample is higher than those of original and recovered SiO_2_/SMPU samples. This is because the molecular chains are stretched from naturally curled state during the programming so that they are elongated. SiO_2_ nanoparticles are moved along with molecular chains to fill the pores in SMPU matrix, which are wrapped by SMPU to increase the specific surface area of porous SMPU. Therefore, the molecules in SMPU which absorb infrared radiation are increased, leading to the energy level transition due to stronger molecular vibration and rotation [47].

As shown in Figure 9a, there are no differences between A**_∥_** and A**_⊥_** of the above characteristic absorption peaks of original SiO_2_/SMPU although SMPU is a typical two-phase structure, indicating the original SiO_2_/SMPU sample shows obvious isotropy, and no orientation occurs in SiO_2_/SMPU. The hard segment acts as a stationary phase, and the molecular chain of soft segment is fixed. The molecular chain of soft segment is naturally curled around hard segment, showing a disorderly arrangement like a tassel shape.

However, it is noted from Figure 9b that A**_∥_** of the above characteristic absorption peaks of hard and soft phases are obviously higher than A**_⊥_** of programmed SiO_2_/SMPU nanocomposite. This indicates that SiO_2_/SMPU shows obvious anisotropy. The reason is that molecular chains of soft segment in SMPU are oriented from naturally curled state along the stretching direction during the programming. Although soft segment, as a reversible phase, does not reach the fully oriented state, it is still changed from the disordered arrangement to the long straight molecular chain morphology, reflecting its anisotropy. 

From Figure 9c, it is found that A**_∥_** and A**_⊥_** of the above characteristic absorption peaks of recovered SiO_2_/SMPU are very close, indicating the programmed SiO_2_/SMPU is almost restored to the isotropic original state after the free recovery. For example, the characteristic peak absorption intensity of soft segment at 2943 cm^−1^ is basically unchanged, but the characteristic peak absorption intensities at 1645 and 1680 cm^−1^ of hard segments are somewhat different. Further, it is also noted from Figure 9a, c that A**_∥_** and A**_⊥_** of the above characteristic absorption peaks of recovered SiO_2_/SMPU nanocomposite are slightly higher those of original sample, suggesting the programmed SiO_2_/SMPU sample is not completely restored to the original state.

The possible reasons are that SiO_2_ nanoparticles and hard phase change their orientations along with the stretching of reversible phase during the programming. As a result, partial hydrogen bonds between hard and soft segments are broken, and some crystal structures are damaged under the action of micro-Brownian motion. Therefore, the programmed SiO_2_/SMPU sample does not completely restored to its original shape.

**Figure 10:**
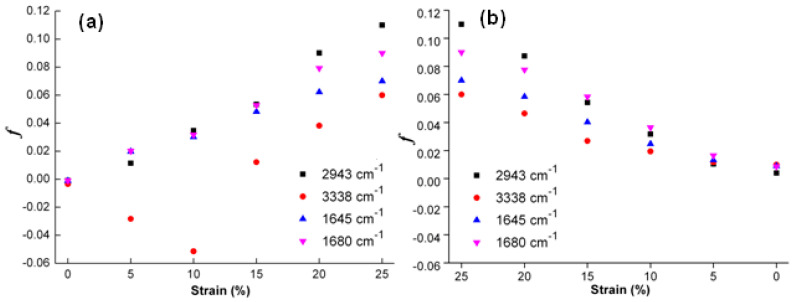
Orientation function value changes at characteristic absorption peaks of SiO_2_/SMPU samples during the (**a**) programming and (**b**) recovery.

To further quantitatively discuss the orientation of main molecular chains, such characteristic absorption peaks at 1645, 1680, 2943 and 3338 cm^−1^ are selected to discuss molecular orientation behaviors of original, programmed and recovered SiO_2_/SMPU samples, respectively [48]. The orientation function values of *f* (1680), *f* (1645), *f* (3338) and *f* (2943) at above characteristic absorption peaks are calculated as shown in Figure 10.

As shown in Figure 10a, such orientation function values as *f* (1680), *f* (1645), *f* (3338) and *f* (2943) at above characteristic absorption peaks of original SiO_2_/SMPU sample are all slightly less than zero. This indicates that some molecular chains of SMPU are perpendicular to the stretching direction at a micro level [49]. It is due to the uniform dispersion of SiO_2_ nanoparticles in SMPU matrix, forming an interfacial region together with soft and hard segments. The –OH groups of SiO_2_ nanoparticles are combined with C=O groups in soft and hard segments to form hydrogen bonds. Therefore, this causes the ordered arrangement of some molecular chains in the original SiO_2_/SMPU sample.

From Figure 10a, as the stretching strain is increased from 0% to 25%, the changing trends of *f* values at all selected characteristic absorption peaks of original, programmed and recovered SiO_2_/SMPU samples are different. Among them, the *f* (2943) value is increased obviously to 0.11 when the stretching strain reaches 25% which is higher than those at other characteristic absorption peaks. This suggests that more obvious orientation occurs in soft segments.

The reasons for this are that the rigidity of hard segment is increased due to the addition of SiO_2_ nanoparticles. This hinders the entanglement between some soft and hard segments, and inhibits the movements of hard segments along the stretching direction during the programming of SiO_2_/SMPU specimen [50]. Thus, the orientation degree of hard segment is significantly lower than that of soft segments, indicating that soft segment is easier to be oriented than hard segment during the programming.

The orientation behavior of hard segments is relatively complicated due to the addition of SiO_2_ nanoparticles. The *f* (1645) and *f* (1680) values are increased with the increase in stretching strain during the programming. This suggests that molecular chains of C=O groups in hard segments are oriented along the stretching direction. This is due to the fact that hydrogen bonds are formed among C=O groups in hard segments, molecular chains in soft segments and SiO_2_ nanoparticles. Thus, molecular chains in hard segments tend to rearrangement as molecular chains of soft segments and SiO_2_ nanoparticles are moved along the stretching direction.

It is also seen from Figure 10a that *f* (3338) value is a negative and its absolute value becomes larger as the stretching strain is increased from 0% to 10%. The possible reason is that molecular chains in soft segments which wrap around N–H molecular chains are orderly rearranged along the stretching direction so that partial hydrogen bonds between molecular chains in soft segments and N–H molecular chains in hard segments are broken [51]. However, N–H molecular chains are still perpendicular to the stretching direction due to its stability, leading to the increase in *f* (3338) absolute value.

Further, some new hydrogen bonds are formed between molecular chains of N–H and C=O groups in hard segments when the stretching strain is increased from 10% to 25%. As a result, N–H molecular chains are also oriented along the stretching direction as C=O molecular chains in hard segments are orderly rearranged [52]. These results of *f* values are consistent with the changes of hydrogen bond indexes of R_N–H_ and R_C=O_ in Table 2. This indicates that molecular chains are oriented during the programming, and some new hydrogen bonds are formed, causing the microphase separation in SMPU to change [53].

As shown in Figure 10b, it is seen that *f* (2943) value of soft segment in recovered SiO_2_/SMPU nanocomposite is almost the same with those of original sample. However, *f* (1645), *f* (1680) and *f* (3338) values of hard segments in recovered SiO_2_/SMPU nanocomposite are slightly larger than those of original SiO_2_/SMPU sample.

This is due to the fact that hard segment is regularly arranged to form microcrystalline or para crystalline structures [54]. When SiO_2_/SMPU specimen is stretched to the strain of 25% during the programming, SiO_2_ nanoparticles are filled in the pores of porous SMPU along with the movement of hard segment to simultaneously extrude some lamella crystals. This facilitates lamella crystals to be oriented along the stretching direction and partial lamella crystals are damaged at the same time so that programmed SiO_2_/SMPU specimen is not completely restored to its original shape. Therefore, this also explains the reason why R*_f_* and R*_r_* cannot reach 100% at a microscopic level as shown in Table 1.

## 4. Conclusions

In this study, SiO_2_/SMPU nanocomposite is prepared, and its thermal behaviors and shape memory performance are characterized. Changes in microstructure, interaction among microphase structures, and molecular orientation of SiO_2_/SMPU are discussed during a five-step thermodynamic cycle. Main conclusions are obtained as follows.

1. The phase separation temperature (72.9 °C) is regarded as the best shape memory switching temperature of prepared SiO_2_/SMPU nanocomposite, which matches the highest working temperature of expansion joints on concrete pavement in China.
2. The SiO_2_ nano particles with an average size of about 70–180 nm could be well dispersed in SMPU matrix and the addition of SiO_2_ nano particles has quite small influence on the thermal behavior and microstructure of SMPU.
3. The shape memory performance of SMPU is affected by SiO_2_ nano particles, in the one shape memory cycle, the average values of R*_f_* and R*_r_* of SiO_2_/SMPU are 98.2% and 97.3%, respectively, showing excellent shape memory effect although crystal imperfections are increased due to the addition of SiO_2_ nanoparticles.
4. The peculiar orientation behaviors of SiO_2_/SMPU nanocomposite in shape memory process could be ascribed to the interactions between SiO_2_ nano particles and segments of SMPU. In programming process, the hard and soft segments at the surface have a perpendicular direction at the small strain, and then possess a parallel orientation at higher deformation.
5. Calculation results of orientation functions suggest that the tensile programming leads to the molecular orientation in SiO_2_/SMPU nanocomposite, showing obvious anisotropy. The programmed specimen is not completely recovered to the original shape because partial hydrogen bonds between hard and soft segments are broken, and some crystal structures are also damaged.

For the next phase, based on the influence of SiO_2_ nano particles on shape memory performance, further study of mechanical properties such as durability will be considered, and multiple shape memory programming of SiO_2_/SMPU nanocomposite will be carried out in combination with actual application scenarios and the optimization of materials to have better self-healing properties.

## Figures and Tables

**Table 1:** Test results of pure SMPU and SiO_2_/SMPU specimen lengths in the programming and free recovery process and calculated results of R*_f_* and R*_r_*.

Sample	*l*_0_ (mm)	*l*_1_ (mm)	*l*_2_ (mm)	*l*_3_ (mm)	R*_f_* (%)	R*_r_* (%)
SMPU	44.00	55.00	55.00	44.00	100	100
	44.00	55.02	55.02	44.00	100	100
	44.00	55.00	55.00	44.00	100	100
SiO_2_/SMPU	44.00	55.03	54.83	44.31	98.18	97.14
	44.00	54.99	54.80	44.29	98.18	97.29
	44.00	54.98	54.76	44.27	98.09	97.49

**Table 2:** Hydrogen-bonding indexes of N-H and C=O functional groups in SMPU at different tensile strain levels during the programming.

Stretching Strains	0%	5%	10%	15%	20%	25%
R_N-H_	0.42	0.42	0.41	0.39	0.38	0.38
R_C=O_	1.12	1.13	1.16	1.19	1.23	1.28

## Data Availability

Not applicable.

## References

[1] Xie Y., Mo L., Su D., Woldekidan M., Wu S. (2013). Investigation into fundamental properties of bituminous plug expansion joint filling mixtures containing rubber granules. Constr. Build. Mater..

[2] Li G., Ji G., Meng H. (2014). Shape Memory Polymer-Based Sealant for a Compression Sealed Joint. J. Mater. Civ. Eng..

[3] Liu S., Mo L., Wang K., Xie Y., Woldekidan M. (2016). Preparation, microstructure and rheological properties of asphalt sealants for bridge expansion joints. Constr. Build. Mater..

[4] Li Q., Crowley R.W., Bloomquist D.B., Roque R. (2014). Newly Developed Adhesive Strength Test for Measuring the Strength of Sealant between Joints of Concrete Pavement. J. Mater. Civ. Eng..

[5] Zhou X.X. (2019). Thermo kinetics study of degradation process of soybean-based polyurethane foams. J. Appl. Polym. Sci..

[6] Hu J.L., Zhang C.L., Ji F.L. (2016). Revealing the morphological architecture of a shape memory polyurethane by simulation. Sci. Rep..

[7] Ban J., Zhu L., Chen S. (2017). The effect of liquid crystal fillers on structure and properties of liquid crystalline shape memory polyurethane composites II: 4-hexadecyloxybenzoic acid. J. Mater. Sci..

[8] Martins G.S., Pereira I.M., Oréfice R.L. (2018). Toughening brittle polymers with shape memory polymers. Polymer.

[9] Ren H., Mei Z., Chen S., Zhuo H., Chen S., Yang H., Zuo J., Ge Z. (2016). A new strategy for designing multifunctional shape memory polymers with amine-containing polyurethanes. J. Mater. Sci..

[10] Chen J., Li X., Zhu Y., Jiang W., Fu Y. (2015). Storable silicon/shape memory polyurethane hybrid sols prepared by a facile synthesis process and their application to aramid fibers. J. Sol-Gel Sci. Technol..

[11] Zhang J., Xu W., Heng K., Chu M., Qian B., Tang J., Liu Z., Huang F. (2022). Dual-Control Mechanism of Water and Temperature in Automatically Pro-grammable Shape Memory Polymers. Macromol. Mater. Eng..

[12] Wongsamut C., Suwanpreedee R., Manuspiya H. (2020). Thermoplastic polyurethane-based polycarbonate diol hot melt adhesives: The effect of hard-soft segment ratio on adhesion properties. Int. J. Adhes. Adhes..

[13] Gupta P., Garg H., Mohanty J., Kumar B. (2020). Excellent memory performance of poly (1,6-hexanediol adipate) based shape memory polyurethane filament over a range of thermo-mechanical parameters. J. Polym. Res..

[14] Turan D., Gunes G., Güner F.S. (2016). Synthesis, Characterization and O_2_ Permeability of Shape Memory Polyurethane Films for Fresh Produce Packaging. Packag. Technol. Sci..

[15] Morozov I.A., Kamenetskikh A.S., Beliaev A.Y., Scherban M.G., Kiselkov D.M., Lemkina L.M. (2021). Physical-mechanical and structural properties of phase-separated rated polyurethane surface treated in argon plasma. Mater. Phys. Mech..

[16] Gonzalez Bertran J., Ardanuy Raso M., Gonzalez Colominas M., Rodriguez R., Jovancic P. (2022). Polyurethane shape memory filament yarns: Melt spinning, carbon-based reinforcement, and characterization. Text. Res. J..

[17] Shi S., Shen D., Xu T. (2017). Microstructural and mechanical property evolutions of shape memory polyurethane during a thermodynamic cycle. J. Appl. Polym. Sci..

[18] Gholami M., Haddadi-Asl V., Jouibari I.S. (2022). A review on microphase separation measurement techniques for polyurethanes. J. Plast. Film. Sheeting.

[19] Shi S., Xu T., Wang D., Oeser M. (2020). The Difference in Molecular Orientation and Interphase Structure of SiO_2_/Shape Memory Polyurethane in Original, Programmed and Recovered States during Shape Memory Process. Polymers.

[20] Zhang L., Kong H., Qiao M., Ding X., Yu M. (2019). Growing nano-SiO_2_ on the surface of aramid fibers assisted by supercritical CO_2_ to enhance the thermal stability, interfacial shear strength, and uv resistance. Polymers.

[21] Yousefi E., Ghadimi M.R., Amirpoor S., Dolati A. (2018). Preparation of new superhydrophobic and highly oleophobic polyurethane coating with enhanced mechanical durability. Appl. Surf. Sci..

[22] Huang M.M., Dong X., Gao Y.Y. (2014). Probing the structure evolution/orientation induced by interaction between polyurethane segments and SiO_2_ surface in shape memory process. Polymer.

[23] Güler G., Vorob’Ev M.M., Vogel V., Mäntele W. (2016). Proteolytically-induced changes of secondary structural protein conformation of bovine serum albumin monitored by Fourier transform infrared (FT-IR) and UV-circular dichroism spectroscopy. Spectrochim. Acta Part A Mol. Biomol. Spectrosc..

[24] Aslan S., Kaplan S. (2018). Thermomechanical and Shape Memory Performances of Thermo-sensitive Polyurethane Fibers. Fibers Polym..

[25] Luo H.S., Zhou X.D., Ma Y.Y. (2016). Shape memory-based tunable resistivity of polymer composites. Appl. Surf. Sci..

[26] Sui T., Salvati E., Zhang H., Dolbnya I., Korsunsky A. (2019). Multiscale synchrotron scattering studies of the temperature-dependent changes in the structure and deformation response of a thermoplastic polyurethane elastomer. Mater. Today Adv..

[27] Cao F., Jana S.C. (2007). Nanoclay-tethered shape memory polyurethane nanocomposites. Polymer.

[28] Armstrong S.R., Du J., Baer E. (2014). Co- extruded multilayer shape memory materials: Nano-scale phenomena. Polymer.

[29] Zhao Q., Qi H.J., Xie T. (2015). Recent progress in shape memory polymer: New behavior, enabling materials, and mechanistic understanding. Prog. Polym. Sci..

[30] Mondal S. (2021). Temperature responsive shape memory polyurethanes. Polym. Technol. Mater..

[31] Luo L., Yuan Y., Dai Y., Cheng Z., Wang X., Liu X. (2018). The novel high performance aramid fibers containing benzimidazole moieties and chloride substitutions. Mater. Des..

[32] Luo L., Wang Y., Dai Y. (2018). The introduction of asymmetric heterocyclic units into poly (p-phenylene terephthalamide) and its effect on microstructure, interactions and properties. J. Mater. Sci..

[33] Mattia J., Painter P. (2007). A Comparison of Hydrogen Bonding and Order in a Polyurethane and Poly(urethane-urea) and Their Blends with Poly (ethylene glycol). Macromolecules.

[34] Tereshatov V.V., Makarova M., Senichev V.Y., Volkova E., Vnutskikh Z.A., Slobodinyuk A.I. (2014). The role of the soft phase in the hardening effect and the rate dependence of the ultimate physico-mechanical properties of urethane-containing segmented elastomers. Colloid Polym. Sci..

[35] Lu X.-L., Lü X.-Q., Wang J.-Y., Sun Z.-J., Tong Y.-X. (2013). Preparation and shape memory properties of TiO2/PLCL biodegradable polymer nanocomposites. Trans. Nonferrous Met. Soc. China.

[36] Jin J., Ma T., Zhang Y. (2016). Chemically inhomogeneous RE-Fe-B permanent magnets with high Figure of merit: Solution to global rare earth criticality. Sci. Rep..

[37] Shiryaev A.A., Voloshchuk A.M., Volkov V.V., Averin A.A., Artamonova S.D. (2017). Nanoporous active carbons at ambient conditions: A comparative study using X-ray scattering and diffraction, Raman spectroscopy and N2 adsorption. J. Phys. Conf. Ser..

[38] Hu J., Wu Y., Zhang C., Tang B.Z., Chen S. (2017). Self-adaptive water vapor permeability and its hydrogen bonding switches of bio-inspired polymer thin films. Mater. Chem. Front..

[39] Zhang S., Ran Q., Fu Q., Gu Y. (2018). Preparation of Transparent and Flexible Shape Memory Polybenzoxazine Film through Chemical Structure Manipulation and Hydrogen Bonding Control. Macromolecules.

[40] Liu W.K., Zhao Y., Wang R. (2017). Post-crosslinked polyurethanes with excellent shape memory property, Macromol. Rapid Commun..

[41] Li W., Jiang X., Wu R., Wang W. (2017). Fast shape recovery by changing the grafting ratio in polyurethane/montmorillonite–poly(methyl methacrylate) composites. Polym. J..

[42] Yu J., Xia H., Teramoto A., Ni Q.-Q. (2018). The effect of hydroxyapatite nanoparticles on mechanical behavior and biological performance of porous shape memory polyurethane scaffolds. J. Biomed. Mater. Res. Part A.

[43] Zhang S., Chen J., Han D., Feng Y., Shen C., Chang C., Song Z. (2015). The effect of soft segment on the microstructure and mechanical properties of waterborne UV-curable polyurethane/silica nanocomposites. J. Polym. Res..

[44] Huang M., Zheng L., Wang L., Dong X., Gao X., Li C., Wang D. (2017). Double Crystalline Multiblock Copolymers with Controlling Microstructure for High Shape Memory Fixity and Recovery. ACS Appl. Mater. Interfaces.

[45] Wang R., Zhang F., Lin W., Liu W., Li J., Luo F., Wang Y., Tan H. (2018). Shape Memory Properties and Enzymatic Degradability of Poly(ε-caprolactone)-Based Polyurethane Urea Containing Phenylalanine-Derived Chain Extender. Macromol. Biosci..

[46] Zhang Y., Li W., Wu R., Wang W. (2017). PU/PMMA composites synthesized by reaction-induced phase separation: A general approach to achieve a shape memory effect. RSC Adv..

[47] Cai C., Wei Z., Wang X., Mei C., Fu Y., Zhong W.H. (2018). Novel double-networked polyurethane composites with multi-stimuli responsive functionalities. J. Mater. Chem. A.

[48] Yan Y., Xia H., Qiu Y., Xu Z., Ni Q.-Q. (2018). Shape memory driving thickness-adjustable G@SMPU sponge with ultrahigh carbon loading ratio for excellent microwave shielding performance. Mater. Lett..

[49] Gu L., Cui B., Wu Q.-Y., Yu H. (2016). Bio-based polyurethanes with shape memory behavior at body temperature: Effect of different chain extenders. RSC Adv..

[50] Wu X., Liu L., Fang W., Qiao C., Li T. (2016). Effect of hard segment architecture on shape memory properties of polycaprolactone-based polyurethane containing azobenzene. J. Mater. Sci..

[51] Liu J., Ji H. (2018). Investigation on Infrared Signature of Axisymmetric Vectoring Exhaust System with Infrared Suppressions. J. Thermophys. Heat Transf..

[52] Zhang M., Xie T., Qian X., Zhu Y., Liu X. (2020). Mechanical Properties and Biocompatibility of Ti-doped Diamond-like Carbon Films. ACS Omega.

[53] Hadi M.M., Nesbitt H., Masood H., Sciscione F., Patel S., Ramesh B.S., Emberton M., Callan J.F., MacRobert A., McHale A.P. (2021). Investigating the performance of a novel pH and cathepsin B sensitive, stimulus-responsive nanoparticle for optimised sonodynamic therapy in prostate cancer. J. Control Release.

[54] Dong Y., Fu Y., Ni Q.-Q. (2015). In-situgrown silica/water-borne epoxy shape memory composite foams prepared without blowing agent addition. J. Appl. Polym. Sci..

